# Epidemiology, Diagnostics and Therapy of Vulvovaginal Fungal Infections

**DOI:** 10.3390/pathogens15090914

**Published:** 2026-08-31

**Authors:** Magdalena Frej-Mądrzak, Agnieszka Jama-Kmiecik, Aleksandra Górzyńska, Nikola Wahdan, Urszula Nawrot

**Affiliations:** 1 Department of Microbiology, Faculty of Medicine, Wroclaw Medical University, Chalubińskiego 4, 50-368 Wroclaw, Poland; magdalena.frej-madrzak@umw.edu.pl; 2 Division of Medical Biology, Faculty of Nursing and Midwifery, Wroclaw Medical University, Chalubińskiego 4, 50-368 Wroclaw, Poland; agnieszka.jama-kmiecik@umw.edu.pl; 3 Department of Pharmaceutical Microbiology and Parasitology, Faculty of Pharmacy, Wroclaw Medical University, Borowska 211a, 50-556 Wroclaw, Poland; aleksandra.gorzynska@student.umw.edu.pl (A.G.);

**Keywords:** vulvovaginal candidiasis, *Candida albicans*, epidemiology, VVC

## Abstract

Vulvovaginal candidiasis (VVC) is one of the most common superficial fungal infections affecting women. A particularly challenging issue is the treatment of recurrent vulvovaginal candidiasis (RVVC)—defined as ≥4 episodes of VVC within 12 months—which affects 5–8% of women with VVC. The development of VVC is influenced by many risk factors related to the host organism and behaviour, including disturbances in the vaginal microflora, hormonal fluctuations, diabetes, hygiene and sexual behaviour. Although *C. albicans* remains the primary species causing this infection, infections caused by other species, such as *Nakaseomyces glabratus* and *Pichia kudriavzevii*, are becoming increasingly common, partly due to the widespread use of azole antifungal drugs and growing drug resistance. Therefore, this publication focuses on a review of the newest literature concerning the epidemiology, diagnosis, current approaches to antifungal susceptibility testing and treatment according to global guidelines. According to the literature, precise species identification and drug susceptibility testing are crucial to avoid therapeutic errors and drug resistance. Moreover, new antifungal drugs such as tetrazoles (oteseconazole) and triterpenoids (ibrexafungerp) are entering clinical practice, offering high efficacy against resistant strains. Probiotics used as adjuvant therapy help restore the homeostasis of the vaginal microbiome; however, due to inconsistent evidence, they are currently not included in treatment recommendations.

## 1. Introduction

*Candida albicans* is the most common species of yeast-like fungi, found in the microbiota of over 70% of the general human population. It colonizes the skin and mucous membranes of the gastrointestinal tract and genital organs [1]. Infections caused by *Candida* species are usually opportunistic. They occur primarily in the presence of tissue disruption, microbiota imbalances, and acquired or congenital immune deficiencies. These infections can be divided into two subtypes: superficial (involving the mucosa and/or skin) and deep (organ-specific and disseminated/systemic). The former includes vulvovaginal candidiasis (VVC), oropharyngeal candidiasis, oesophageal candidiasis, and candidiasis of the nails and skin, and is found in both individuals with a normal and compromised immune system. People with severe immune deficiencies (e.g., long-term neutropenia) are particularly susceptible to severe *Candida* infections, which may take the form of fungaemia, hepatosplenic abscesses, myocarditis, central nervous system inflammation, arthritis, or, less frequently, lung infections [2]. Deep candidiasis may develop as a result of exogenous infection, following tissue disruption due to trauma or invasive medical procedures (vascular catheters, surgery). It is believed that antifungal prophylaxis used in some at-risk patients, e.g., Haematopoietic Stem Cell Transplant (HSCT) patients, neutropenic patients, critically ill patients, post-abdominal surgery patients, and particularly long-term use of azole drugs, contributes to an increased prevalence of infections involving species naturally more resistant to these drugs, such as *Nakaseomyces glabratus* (formerly *Candida glabrata*) or *Pichia kudriavzevii* (formerly *Candida krusei*), while simultaneously reducing the percentage of infections caused by *C. albicans*.

The subject of this review is genital candidiasis. Although the topic has been widely described in the literature, there are still some knowledge gaps in this area, warranting further attention. In this narrative review, the authors present the most recent publications and recommendations to shed light on this problem. These infections are usually limited to the vagina and vulva and are characterized by inflammation accompanied by redness and itching of the vaginal and vulvar mucosa, burning, and cheese-like discharge. In some patients, VVC may be recurrent. Recurrent vulvovaginal candidiasis (RVVC) is defined as ≥4 episodes of VVC within 12 months [3]. Another form of VVC, distinguished by some authors, is chronic vulvovaginal candidiasis (cVVC), which manifests as prolonged vaginal inflammation without asymptomatic periods, as is the case with RVVC [4]. The main reservoir of *Candida* spp. is the lower gastrointestinal tract, from which the fungi can easily spread to the skin around the perineum and genitals, as well as the oral cavity. Vaginal mycoses are most often endogenous infections, but the fungi can also be sexually transmitted. Genital mycoses can also affect men, taking the form of candidal balanitis, although this is a less common problem, and antifungal therapy for sexual partners is not always implemented. It is estimated that VVC affects 70–75% of women at least once during their lifetime, with 40–50% experiencing recurrence; in 5–8% of cases, these infections occur at least four times per year. Although the infection may occur at any age, it is particularly common during reproductive age, less common in postmenopausal women, and only occasionally reported in children [3,4,5,6]. These infections do not pose a direct threat to life and are therefore often downplayed. However, they are certainly a global problem, reducing quality of life and potentially contributing to the development of depression in women they affect. At the same time, they have a significant economic impact. There is an enormous demand for topical and systemic medications and supplements used in the treatment/prevention of VVC, which is why they constitute an important branch of production in the pharmaceutical industry. The clinical presentation of vaginal mycosis and the physical examination (redness of the mucosa, itching, cheese-like discharge) are so characteristic that they are often considered sufficient criteria for initiating therapy. Moreover, patients with a history of infection sometimes self-diagnose based on subjective assessment of symptoms and initiate antifungal therapy, which in many countries does not require a doctor’s prescription. This approach carries the risk of misdiagnosis (non-infectious diseases, e.g., allergies, infections of other etiologies: bacterial vaginosis, viral infection, trichomoniasis, etc.), overuse of antifungal medications, or incorrect dosage and duration of therapy. Errors in therapy may contribute to the development of strains resistant or tolerant to antifungal drugs. Another consequence is the distortion of epidemiological data regarding the incidence of vaginal mycosis and its etiological factors [6,7].

## 2. Risk Factors for VVC

The most common host factors predisposing to the development of vulvovaginal mycoses are changes in the composition of the vaginal microbiota, hormonal imbalances, and diabetes. Behavioural factors such as insufficient or excessive hygiene, sexual activity, frequent changes in sexual partners, oral-genital sex, etc., also contribute to the occurrence of VVC. In a relatively small percentage of women, susceptibility to fungal infection may be genetically determined or associated with weakened immunity due to previous diseases and therapies [3,6].

### 2.1. Dysbiosis

**Figure 1:**
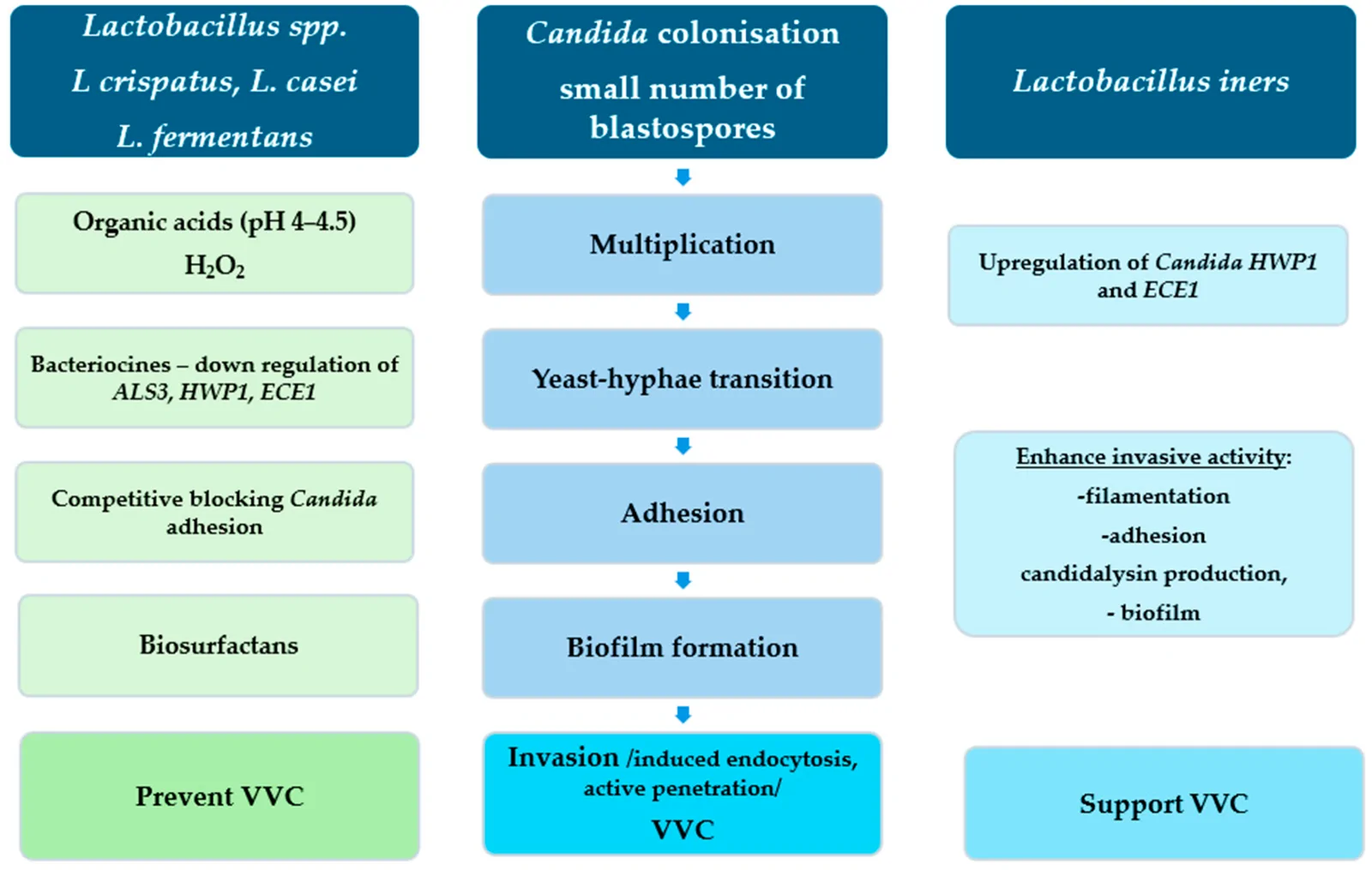
Impact of different *Lactobacillus* spp. on VVC development.

The vaginal microbiome plays a key role in maintaining vaginal health and preventing VVC. In healthy women, it is typically dominated by *Lactobacillus* spp., which help maintain a low pH environment and produce lactic acid, hydrogen peroxide, and antimicrobial compounds—bacteriocins and biosurfactants. Metabolites produced by *Lactobacillus*, including short-chain fatty acids and lactate, have a direct impact on the morphogenesis of *Candida albicans*. This is believed to be one of the most important factors inhibiting the production of invasive forms—hyphae—and, consequently, the development of VVC. In the presence of these bacteria, *C. albicans* colonises the mucosa almost exclusively in the blastospore form [8,9]. The use of broad-spectrum antibiotics (e.g., tetracycline, penicillin) or other factors, such as frequent vaginal douching with antimicrobial intimate hygiene products, may result in complete elimination or a drastic reduction in the number of *Lactobacillus*. This condition, called dysbiosis, promotes the proliferation of *Candida* and the production of invasive forms, which consequently leads to the development of symptomatic infection. In addition to a decrease in the number of *Lactobacillus* spp., vaginal dysbiosis is characterized by an increased proportion of morphologically diverse bacteria [10,11]. Studies of the vaginal microbiota of women with VVC have shown a reduction in *Firmicutes* (mainly *Lactobacillus* sp.) and an increase in *Actinobacteria*, *Bacteroidetes*, and *Proteobacteria* [10]. Furthermore, it was found in vitro that *Lactobacillus iners* acts in the opposite way to other *Lactobacillus* species; it promotes *Candida* hyphae formation, adhesion, the expression of *HWP1* (hypha-associated protein) and *ECE1* (gene encoding candidalysin), as well as strong biofilm production, which could exacerbate the infection (Figure 1) [12]. This hypothesis appears to be supported by recent metagenomic studies indicating a high proportion of *L. iners* in the microbiome of patients with RVVC [13].

Despite numerous studies, the interactions between *Candida* spp. and other microorganisms comprising the vaginal microbiome remain incompletely understood. Much suggests that they may represent an important point of investigation for developing more effective strategies for the prevention and treatment of VVC [12,13,14,15].

### 2.2. Hormonal Changes

An association has been observed between high oestrogen levels and an increased susceptibility to developing vaginal candidiasis in women during puberty, particularly during pregnancy. This increase in hormone levels may be physiological or result from endocrine disorders and therapies, including hormonal contraception and hormone replacement therapy, which are also associated with an increased risk of VVC. In the period before menarche and in postmenopausal women not using hormone replacement therapy, the likelihood of vaginal colonization by *Candida* is reduced due to reduced vaginal oestrogenization [16,17]. Mice are a frequently used animal model in studies on the pathogenesis of VVC, with their susceptibility to developing this infection determined by the administration of exogenous oestrogens. Hormones create an environment conducive to fungal proliferation, affecting both the host and the pathogens. Elevated oestrogen levels stimulate glycogen deposition in the vaginal epithelium, which can serve as a carbon source for yeasts and promote epithelial cornification and keratinisation. It also influences the immune response, limits leukocyte infiltration, and reduces local defence mechanisms in the vaginal mucosa. *Candida albicans* cells have been shown to possess receptors that bind oestradiol derivatives, and that hormones enhance morphogenesis (hyphal production) and adhesion of yeast to the vaginal epithelium [18].

### 2.3. Diabetes

Diabetes and unregulated blood sugar levels are known risk factors for bacterial and fungal infections, particularly candidiasis. Susceptibility to infection is associated, among other things, with the negative impact of high glucose levels on the immune system, including T cells, macrophages, neutrophils, and NK (Natural Killer) cells [19,20]. High glucose concentrations are observed not only in the blood but also in mucosal secretions, where they promote the massive proliferation of yeasts, increasing their adhesion and biofilm formation, which directly promotes the development of infection. Patients with unregulated diabetes have a significantly higher incidence of vulvovaginal candidiasis, including RVVC [20,21]. The most important data on factors influencing persistence of *Candida* on vaginal mucosa are summarised in Table 1.

### 2.4. Genetic Predisposition and Immune Deficiencies

Vaginal candidiasis is one of the infections that occurs in patients with certain congenital and acquired immune system disorders (Dectin-1 receptor defects, TLR2 polymorphism, IL-17 and IFN-γ deficiency, immunosuppressive therapy), which, among other factors, increase the risk of recurrent and chronic fungal infections [6]. Susceptibility to RVVC has been observed to be higher in certain ethnic groups (e.g., African Americans) and correlated with polymorphisms of certain genes, such as the mannose-binding lectin 2 (MBL2) gene [3]. Cases of RVVC most often affect women without severe immune deficiencies and otherwise considered healthy. For decades, scientists have been trying to understand this phenomenon, and a more comprehensive analysis of this issue has recently been published [3,22]. One recent hypothesis points to neutrophil (PMN) anergy as the likely cause of symptomatic infection. The pathophysiology of vaginal candidiasis is known to involve the adhesion and invasion of the vaginal mucosa by fungi and a strong local inflammatory response. Despite the presence of cells with fungicidal activity (polymorphonuclear neutrophils (PMNs) and vaginal epithelial cells), fungi are not eliminated in patients with cVVC. Examining PMN activity in cVVC-susceptible mice, Yano et al. detected the proteoglycan HS (heparan sulfate) in the vaginal secretions of these animals. This compound was found to be a ligand binding the Mac-1 receptor, which is essential for the recognition and killing of fungi by neutrophils. The blocking of fungicidal activity and functional anergy of PMNs caused by HS proteoglycan may contribute to the occurrence of chronic forms of VVC [23].

## 3. Etiology of VVC

As mentioned earlier, the most common etiological factor of uncomplicated VVC, accounting for 80–90% of cases, is *Candida albicans*. This species also predominates in patients with RVVC, but in a much lower percentage (30–60%). Approximately half of RVVC cases and 10–20% of VVC cases are caused by non-*Candida albicans* yeast (NCAY) species, such as *Nakaseomyces glabratus*, *Candida tropicalis*, *Candida parapsilosis*, or *Pichia kudriavzevii,* as well as species/strains closely related to *C. albicans*, such as *C. africana* and *C. dubliniensis* [4,7,8]. In some studies, the so-called true yeast or baker’s yeast, *Saccharomyces cerevisiae*, is reported as an etiological factor of up to 3–10% of vaginal and vulvar infections due to NCAY [24,25,26]. This may pose a particular problem because *Saccharomyces cerevisiae* shares morphological characteristics with many species of the genus *Candida*, and, for this reason, is often misidentified. Furthermore, clinical isolates are more likely to exhibit resistance to azoles, particularly fluconazole, than *C. albicans* [25,27]. Infections caused by NCAY species may be mildly symptomatic and often respond less well to standard therapies. Mixed infections, e.g., between *C. albicans* and *N. glabratus*, are also observed, where the more virulent species (*C. albicans*) paves the way for the less virulent species (*N. glabratus*), which is incapable of invading the host’s healthy mucosa. The different species composition of recurrent infections is likely related to the selective pressure of azoles, which are used more frequently in patients with RVVC than in those treated for episodic VVC. It should be added that basidiomycete yeasts from the genera *Rhodotorula*, *Cryptococcus*, and *Trichosporon* are occasionally isolated from vaginal infections, and even molds (e.g., *Mucor*) are occasionally isolated [28,29]. There is no consensus within the medical community regarding the actual pathogenic potential of rare NCAY (especially basidiomycete yeasts). According to experts, in most cases they are part of the microbiome rather than pathogens responsible for infection, as they (e.g., *Rhodotorula* spp) have often been isolated from the vulva and vagina of asymptomatic patients. A lack of response to the prescribed treatment may indicate not so much strain drug-resistance as the presence of another, non-fungal cause of the clinical symptoms and requires further diagnostics. It is worth noting that, according to the classification introduced by the CDC (Centers for Disease Control), vulvovaginal candidiasis caused by non-albicans *Candida* species, along with recurrent and chronic infections, as well as VVC in patients with predisposing factors (including mucocutaneous candidiasis) and a severe clinical course, are classified as “complicated VVC”. This implies a higher risk of treatment failure, especially after a standard course of azole therapy, and the need for prolonged treatment [26,30].

### 3.1. Why Is Candida albicans the Most Common?

*Candida albicans* is the best-known fungal pathogen. It is undoubtedly a microorganism perfectly adapted to the human body, capable of growing both as a commensal and as a pathogen. Over the course of evolution, it has developed a range of mechanisms allowing it to survive in changing environmental conditions, including metabolic adaptation, drug tolerance, morphological and antigenic variability, and the ability to evade and modulate host defense mechanisms [31,32,33]. Its pathogenic properties result from the presence of several virulence factors, such as hyphal-yeast dimorphism, phenotypic switching (white-opaque switching), expression of surface adhesins and invasins, biofilm formation, secretion of hydrolytic enzymes and candidalysin, and the ability to invade and damage host cells. Animal studies have shown that *C. albicans* mutants lacking the ability to form hyphae are characterised by reduced or complete loss of virulence [32]. This transformation determines the pathogen’s invasiveness, causes resistance to macrophages, enhances cell adhesion, and biofilm formation [33]. The change in morphology can be induced by environmental conditions. In vitro studies have shown that hyphal formation is favored by a temperature of 37 °C, neutral pH, increased CO_2_ concentration, the presence of serum, and N-acetylglucosamine. Blastospores are observed primarily at acidic pH and lower temperatures (approximately 30 °C), while pseudohyphae are observed in intermediate conditions [34]. Vaginal infection typically develops when yeast outgrows the bacterial microbiota, making epithelial cell receptors more accessible to blastospores. Blastospore adhesion, primarily mediated by the Als 5 adhesin (from the agglutinin-like sequence family) and β-1,3-glucan, signals hyphal germination. These structures are characterised by thigmotropism, manifested by the movement of cells along the epithelium. The hyphae expose proteins specific to this morphological form (e.g., Hwp1—hyphal-specific cell wall protein) that bind to appropriate epithelial cell receptors. Molecular studies have shown that the vaginal secretions of women with VVC highly express genes encoding adhesins Als1, Als2, Als3, and Als9 from the agglutinin-like sequence family [33,35]. The adhesins Als 3 and Sss1, which bind to E-cadherin of epithelial cells, initiate the processes of endocytosis and internalisation of the fungus. In turn, hydrolytic enzymes (secreted aspartyl proteases Sap, phospholipases, lipases) released by apically growing hyphae, as well as the cytolytic exotoxin candidalysin, contribute to the degradation of cell membranes and enable the spread of infection to other cells and adjacent tissues through active penetration [31,32,33,34,35]. Candidalysin also exhibits dual functions in its interaction with the immune system. Produced by fungi germinating within phagocytes, it causes damage to the immune cell membrane through intercalation, permeabilisation, and pore formation, facilitating fungal escape from phagosomes. It also exhibits immunostimulatory effects, promoting cytokine influx into the epithelium and activating IL-1β, IL-36, and the NLRP3 inflammasome, thereby inducing a proinflammatory response and polymorphonuclear leukocyte migration [36,37,38,39]. The polymorphism of *Candida albicans* is also reflected in the complex structure of the biofilms it forms. A biofilm is defined as a highly organised, interconnected structure composed of microorganisms, bound to a substrate or to each other, and embedded in a matrix composed of extracellular polymeric substances (EPS) [40]. This common form of microbial life is considered a significant component of their pathogenicity. This is due to the natural characteristics of biofilms, such as increased tolerance/resistance to drugs, antiseptics, and the immune system, as well as the ability to spread (disperse), i.e., release cells exhibiting the biofilm phenotype, including persisters. In specific clinical situations (e.g., infected vascular catheters), this mechanism can lead to pathogen dissemination into the bloodstream and catheter-related infections. The process of biofilm formation by *Candida* is initiated by blastospores, which attach to the surface of the biomaterial/tissue and multiply, forming a basal layer. Subsequently, filamentous forms (hyphae and pseudohyphae) develop, which rise above the basal layer and form a multilayered network. During the maturation phase, the resulting structure is covered with a matrix composed of exopolymers, which determines the stability of the network. The biofilm matrix consists of carbohydrates, proteins, lipids, and DNA [40,41]. This biofilm structure facilitates the influx of nutrients, the spread of infection, and its resistance to treatment. The extracellular matrix acts as a physical barrier to the drug and strengthens the integrity of the entire structure. One of the components contributing to the barrier nature of the matrix is β-1,3-glucan. This is evidenced by studies demonstrating that treating biofilm-associated infections with β-1,3-glucanase increases their sensitivity to fluconazole, while the addition of exogenous β-1,3-glucansincreases the tolerance of planktonic cells to this drug. Another important component is extracellular DNA. Administration of DNA-ase has been shown to increase the activity of caspofungin and amphotericin B against mature biofilms [41,42]. During the dispersion stage, yeast cells are continuously released (detachment from biofilm) from the top-most hyphal layers of the biofilm, thus spreading the pathogen to new sites of infection. The released cells are characterised by an elongated shape, low metabolic activity, and significant resistance to antifungal drugs and the host’s immune response (phagocytosis) [40,41,43,44,45]. The tolerance of mature biofilms to drugs, particularly azoles such as fluconazole, is not associated with permanent genetic changes but rather results from the accumulation of a series of adaptive changes. Cell membranes in mature biofilms contain less ergosterol (a target for polyenes and azoles) compared to planktonic cells and young, emerging biofilms. Another characteristic of biofilms is the increased activity of efflux pumps (including CDR1 and CDR2 of the ATP-binding cassette transporter superfamily as well as MDR1 from the major facilitator class), which remain overexpressed regardless of the presence or absence of antifungal drugs [41]. A number of metabolic changes are also observed, including the activation of protein synthesis pathways that determine resistance to oxidative stress (e.g., heat shock proteins—Hsps) or DNA damage response, which, among other things, determines resistance to phagocytosis, stimulates morphogenesis and, as a result, affects the survival and pathogenicity of the microorganism [32,46]. A fascinating feature of biofilm-forming cells is their ability to communicate and coordinately respond to environmental signals using quorum-sensing molecules (QSMs), such as farnesol, tyrosol, phenylethanol, and tryptophan. These compounds influence fungal morphogenesis and apoptosis, as well as the induction of an inflammatory response. QSMs appear to play a role in both virulence and infection progression, as well as in their self-limitation, making them an attractive alternative in the search for new antifungal therapies. Furthermore, synergistic effects have been demonstrated between some QSMs, e.g., farnesol, and numerous antifungal drugs, including fluconazole, micafungin, and amphotericin B [47,48]. The structure of biofilms formed in vivo may differ significantly from that observed under experimental conditions. Their development in vivo is influenced by pathogen–host interactions, including the availability of nutrients, the composition of mucosal secretions, and the presence of the accompanying microbiota. This applies primarily to mucosae, where polymicrobial biofilms can form, resulting from complex antagonistic–synergistic interactions between different microbial species inhabiting a given niche [32]. The presence of *Candida* biofilms during vaginal infection was first demonstrated in an animal model [49]. A recently published study confirmed the presence of epithelium-associated *Candida* biofilms in vaginal samples/biopsies from women with RVVC [50]. This work supports the role of biofilms in the pathogenesis of vaginal candidiasis. The authors hypothesise that the formation of biofilms, resistant to standard antifungal therapies, causes recurrent symptomatic infection, referred to as RVVC.

Among studies on the pathogenesis of candidiasis, those comparing the virulent potential of clinical isolates obtained from different niches and types of infections have been important. Significant variation has been demonstrated in the production of individual virulence factors (adhesion, biofilm, enzyme production, etc.) by individual clinical isolates of *Candida albicans*. This may raise concerns that hypervirulent strains, causing particularly severe infections, will emerge as this species evolves. To date, no strict association has been demonstrated between individual *C. albicans* genotypes and phenotypes and their invasiveness. This also applies to strains causing symptomatic VVC, which exhibit genetic divergence similar to that of colonising strains (MLST-genotype, candidalysin sequence) [51]. In the vaginal secretions of women with VVC, *C. albicans* forms mainly filamentous forms, whereas in colonised women, it forms blastospores. However, it has been shown that invasive and colonised isolates have a similar ability to form hyphae in vitro. A recently published study by Pericolini et al. [51,52] showed that VVC isolates differ from commensal isolates in their response to β-1,3-glucan, a cell wall component responsible for the induction of an inflammatory response. Isolates from infection activate different signalling pathways in epithelial cells; this includes lower activation of the type I interferon pathway, which correlates with increased cytopathic effect. According to the authors, differential induction of epithelial responses, including the interferon pathway, may be used in the future in VVC diagnostics to assess the in vitro pathogenic potential of strains isolated from patients.

Studies of virulence factors of yeast species from the NCAY group have revealed significant differences compared to *C. albicans*. This applies primarily to morphogenesis and invasiveness; with the exception of the species *C. dubliniensis*, *C. tropicalis*, and *P. kudravzevii*, they are unable to produce true hyphae, and some, such as *Nakaseomyces glabratus*, exist only in the form of blastospores. The adhesion and production of extracellular hydrolytic enzymes such as phospholipases and proteases, as well as the thickness of formed biofilms, are usually much weaker than in *C. albicans.* As a result, they rarely cause acute infections; more often, they are present on mucous membranes as part of the microbiome or cause mild, recurrent infections that are difficult to completely eradicate. A distinctive feature of *N. glabratus*—the second most commonly isolated pathogen causing VVC—is its ability to survive inside macrophages, resulting from the inhibition of phagolysosome formation, resistance to ROS (Reactive Oxygen Species), and intracellular killing. Furthermore, in the case of this species, significant differences in the induction of the immune response have been observed; it stimulates the production of granulocyte-macrophage colony-stimulating factor (GM-CSF) and not pro-inflammatory cytokines, which results in the recruitment of macrophages instead of neutrophils. A change in the profile of cytokines produced and weakening of the protective Th1-type response are observed in RVVC cases [53].

### 3.2. Incidence of Vulvovaginal Fungal Infections (VVC)

The incidence of VVC is global and affects women of all races and nationalities. Some studies indicate a higher incidence of VVC in developed countries, which may be due to the increased use of antibiotics, increased use of hormonal contraception, and improved availability of microbiological diagnostics. Although vaginal fungal infections occur in women of all ages, both sexually active and inactive, they pose the greatest problem in women of reproductive age. Groups at risk for VVC include women aged 18–45, pregnant women—especially in the second and third trimesters (up to 30% of pregnant women), women using hormonal contraceptives with high oestrogen content, and women with diabetes, especially those with uncontrolled glycemia, and those with weakened immune systems (HIV-positive, post-transplant, or undergoing immunosuppressive therapy) [14]. A little-known consequence of vaginal candidiasis in pregnancy, or even its asymptomatic colonisation, is the risk of vertical and perinatal infections. In a recently published study on an Ethiopian population, Gedefie et al. [54] demonstrated *Candida* infection in 22/49 (44.9%) newborns born to mothers colonised with the fungus.

VVC is a significant public health problem. It is estimated that more than 6 million cases of VVC occur annually in the United States, of which approximately 1.4 million require medical treatment. Consequently, the costs of treatment and lost productivity in the United States amount to hundreds of millions of dollars annually [17].

According to the study by Denning et al. [55], the estimated burden of RVVC per 100,000 women worldwide is 3871, ranging from <3400 (Timor-Leste, Nigeria, Ethiopia, Congo, Uganda, etc.) to >4300 (Iran, China, Vietnam, etc.). These data are only estimates; as the authors emphasise, knowledge regarding the incidence of VVC/RVVC is incomplete, and population-based studies on unselected populations are lacking. Because the diagnosis is often made empirically, without confirmation by culture, there is a risk of false results (both overestimation and underestimation).

Table 2 summarises selected publications on the incidence of VVC published in the last five years. The percentage of women with VVC ranges from 5% in the general population to several dozen (50%) when studying pregnant women, women with diabetes, or those infected with HIV.

## 4. Diagnostics

*Candida *vaginitis is associated with such clinical symptoms and signs as vulvar pruritus, pain, swelling, redness, and thick curdy vaginal discharge. The presence of the above-mentioned symptoms is an indication to start diagnostic procedures. In addition to a physical gynaecological examination and testing the pH of vaginal discharge (usually normal in VVC and high in bacterial infection), complete microbiological testing should be performed. Classical examination of specimens collected from the genital tract includes direct microscopy (detection of blastospores and/or fungal hyphae) as well as culture followed by species identification and determination of susceptibility to antifungal drugs (Table 3) [26,30,69,70,71,72]. Laboratory diagnosis of VVC appears to be straightforward compared to deep-seated fungal infections; diagnostic specimens can be easily obtained, and the microorganisms causing the disease can be readily cultured in vitro. However, the difficulty increases when it is necessary to distinguish between *Candida* carriage and VVC, or a mixed infection involving more than one yeast species or a combination of fungal, bacterial, parasitic, and viral infection, particularly in previously treated patients with cVVC and RVVC, where the clinical picture may be less obvious. Results should be interpreted with caution, taking into account also the patient’s history. A negative culture result may be related to, for example, “self-medication” applied by patients without informing their physician. Furthermore, diagnosis can be especially challenging in mixed infections when the actual etiological factors are difficult to culture. Therefore, the diagnosis of VVC should include differential diagnosis with other conditions, such as bacterial vaginosis, trichomoniasis, or vulvar dermatoses (e.g., lichen sclerosus, eczema), which may present with symptoms similar to those of candidiasis [26,30,70,71,72]. It should also be noted that symptoms suggestive of VVC may in fact be an allergic reaction to *Candida* antigens—which can be triggered by even a minimal fungal load—or to other allergens unrelated to the presence of these fungi on the vaginal mucosa. Persistence of the infection syndrome may be related, for example, to atopic dermatitis of the vulva, and prolonged antifungal therapy in such a situation will be ineffective [24,26]. An unsatisfactory response to antifungal therapy should prompt a comprehensive diagnostic evaluation [69,70,71,72,73,74].

Over the past decade, many scientific societies—both local and international—have developed and published their own guidelines for the diagnosis and treatment of vaginal candidiasis. In this review, we focused mainly on the 2025 global guidelines for the diagnosis and treatment of candidiasis, developed in collaboration with experts from three leading international societies—the European Confederation of Medical Mycology (ECMM), the International Society for Human and Animal Mycology (ISHAM), and the American Society for Microbiology (ASM) [70], the “Recommendations for the Diagnosis and Treatment of Vaginitis” developed by the International Society for the Study of Vulvovaginal Disease in 2023 [26] and recommendations published on the website of the U.S. Centers for Disease Control and Prevention (CDC) [30].

All these documents recommend microscopic examination of vaginal secretion using wet preparation (0.9% NaCl, 10% KOH) and light or phase-contrast microscopy. Detection of fungal elements (blastospores, hyphae) is enough to start antifungal therapy, whereas negative results could be confirmed by culture. Although the microscopy method is easy, rapid, and does not require advanced technology, it is strongly dependent on the observer’s qualifications and subjective judgment, as well as the quantity of fungal cells present in the sample. To increase the sensitivity of the direct smear, the use of brightening agents (e.g., calcofluor white) and fluorescence microscopy or Gram staining could be used, as this makes it possible to improve visualisation of fungal elements and diminish false results [26,70]. The advantage of Gram-stained preparation is the additional possibility of evaluating the sample not only for the presence of fungi but also bacterial morphotype; however, it is more time-consuming and costly compared with wet preparation.

The culture of vaginal discharge is a gold standard procedure, which is especially helpful in establishing diagnosis in complicated cases of VVC. This method requires an incubation time of at least 24–72 h but gives the possibility of detecting and semi-quantitatively estimating the intensity of fungal growth, as well as subsequent species identification and, if needed, susceptibility testing. The use of chromogenic media facilitates the detection of mixed infections involving several *Candida* species and enables preliminary species identification [17]. Currently, the MALDI-TOF MS method has become the gold standard for the precise and rapid species identification of isolates [70].

An antimycogram is essential, especially in cases of treatment failure and recurrent vaginal yeast infections, due to increasing resistance to azoles, particularly among non-albicans species [71]. Moreover, it can be especially challenging, because commercial tests often do not include topical antifungals (e.g., nystatin or clotrimazole), and even if they do, the clinical breakpoints for those drugs are not established by EUCAST. Another concern is that reference methodology does not reflect vaginal pH conditions, and therefore the results obtained may not reflect in vivo activity of the tested compounds.

Molecular methods, such as PCR, are valuable confirmatory tools; however, their high sensitivity requires careful interpretation in light of clinical symptoms to distinguish an active infection from asymptomatic colonization [69,73]. It is worth remembering that the detection of fungi, whether through classical diagnostics or molecular methods, in asymptomatic women is not an indication for therapy. Despite the obvious potential of nucleic acid amplification-based methods—including the ability to rapidly detect and identify pathogens and even resistance genes—they are not yet sufficiently widely available in VVC diagnostics. Some commercial test kits designed for genital tract infections include fungi in addition to other pathogens. They are sometimes used instead of microscopic examinations when the latter are unavailable. A comprehensive review of rapid molecular VVC diagnostics was recently prepared by Akinosoglou et al. [73]. A brief overview of the methods applied in VVC diagnosis is presented in Table 3.

## 5. Antifungal Drugs and Treatment

Drugs active against yeasts of the genus *Candida* include polyenes, fluorinated pyrimidine derivatives, azoles, and echinocandins. An example of a polyene drug is amphotericin B (AMB). The mechanism of action of this group of drugs involves binding to ergosterol and forming pores, which results in osmotic lysis of the cells. AMB also causes oxidative damage and ergosterol sequestration. However, this drug is associated with a number of side effects, such as high nephrotoxicity, which is greater than that of other polyenes (used primarily for local candidiasis). Resistance to AMB is relatively rare and stems from mutations in genes of the ergosterol synthesis pathway (ERG3, ERG6), which result in reduced sterol concentrations and impaired drug binding. The most commonly used polyenes in the topical treatment of vaginal and vulvar infections are nystatin and natamycin [75].

Flucytosine (5-fluorocytosine, 5-FC) is a drug belonging to the pyrimidine analog class and acts as a nucleic acid inhibitor. After being taken up by *C. albicans*, it is converted to 5-fluorouracil, which disrupts DNA and RNA synthesis. The mechanism of yeast resistance to 5-FC is based on a mutation in the uracil phosphoribosyltransferase gene—an enzyme that prevents the conversion of 5-fluorouracil to 5-fluorouridine monophosphate. Flucytosine is typically administered in combination therapy with AMB or triazoles for invasive infections, particularly those of the central nervous system [40,41]. In vaginal infections caused by azole-resistant species (e.g., *N. glabratus*), 5-FC is sometimes used topically in combination with amphotericin B [69].

Another group of drugs consists of azoles, which are classified based on the number of nitrogen atoms in their ring: imidazoles (e.g., clotrimazole, miconazole) with 2 nitrogen atoms; triazoles with 3 nitrogen atoms (e.g., fluconazole—FLU); and tetrazoles (4 nitrogen atoms), such as oteseconazole. Azoles bind to the iron atom in the heme group of the lanosterol 14α-demethylase enzyme (an enzyme belonging to the cytochrome P450 system, designated CYP51). Inhibition of this enzyme prevents the conversion of lanosterol to ergosterol. These drugs exhibit fungistatic activity against *C. albicans* by inhibiting ergosterol biosynthesis, which leads to the accumulation of toxic sterols and arrest of fungal growth. Resistance to azoles results from mutations in the ERG11 gene and/or its overexpression, increased drug efflux due to the overexpression of efflux pumps (such as CDR1, CDR2, MDR1), or the utilisation of host cholesterol [75]. A mutation in the ERG3 gene halts the production of toxic sterols, resulting in the preservation of membrane structure and rendering the drug ineffective [76]. Oteseconazole, a modern tetrazole-class drug with high selectivity for the fungal enzyme CYP51, a long half-life (138 days) and a drug exposure window of 690 days, was approved by the U.S. Food and Drug Administration (FDA) in July 2022 for the treatment of RVVC. Due to its potential teratogenicity, it must not be used in women who are pregnant or planning to become pregnant. The above-mentioned limitation was the reason for the withdrawal of the oteseconazole application by the European Medicines Agency (EMA), following its initial evaluation of the drug [69,70,77].

Echinocandins (e.g., caspofungin, micafungin, anidulafungin, and the new rezafungin) are antifungal drugs used in the treatment of invasive candidiasis. They act by inhibiting β-1,3-glucansynthase, the main component of the cell wall, which leads to cell lysis [70,75]. Resistance of *Candida* to echinocandins is rare and results from mutations in the β-1,3-glucansynthase subunit encoded by the FKS genes [70,75]. Rezafungin, administered once weekly, has become a new treatment option for candidemia [70].

Among the modern drugs introduced into therapy is ibrexafungerp—the first oral triterpenoid glucan synthase inhibitor, demonstrating high efficacy in azole-resistant cases. In the United States, it is approved for the treatment and prevention of recurrent vaginal candidiasis; in Europe, it has orphan drug status for the treatment of invasive candidiasis (12 November 2021). Due to its potential embryotoxic effects, it must not be used in pregnant women [69,70].

**Figure 2:**
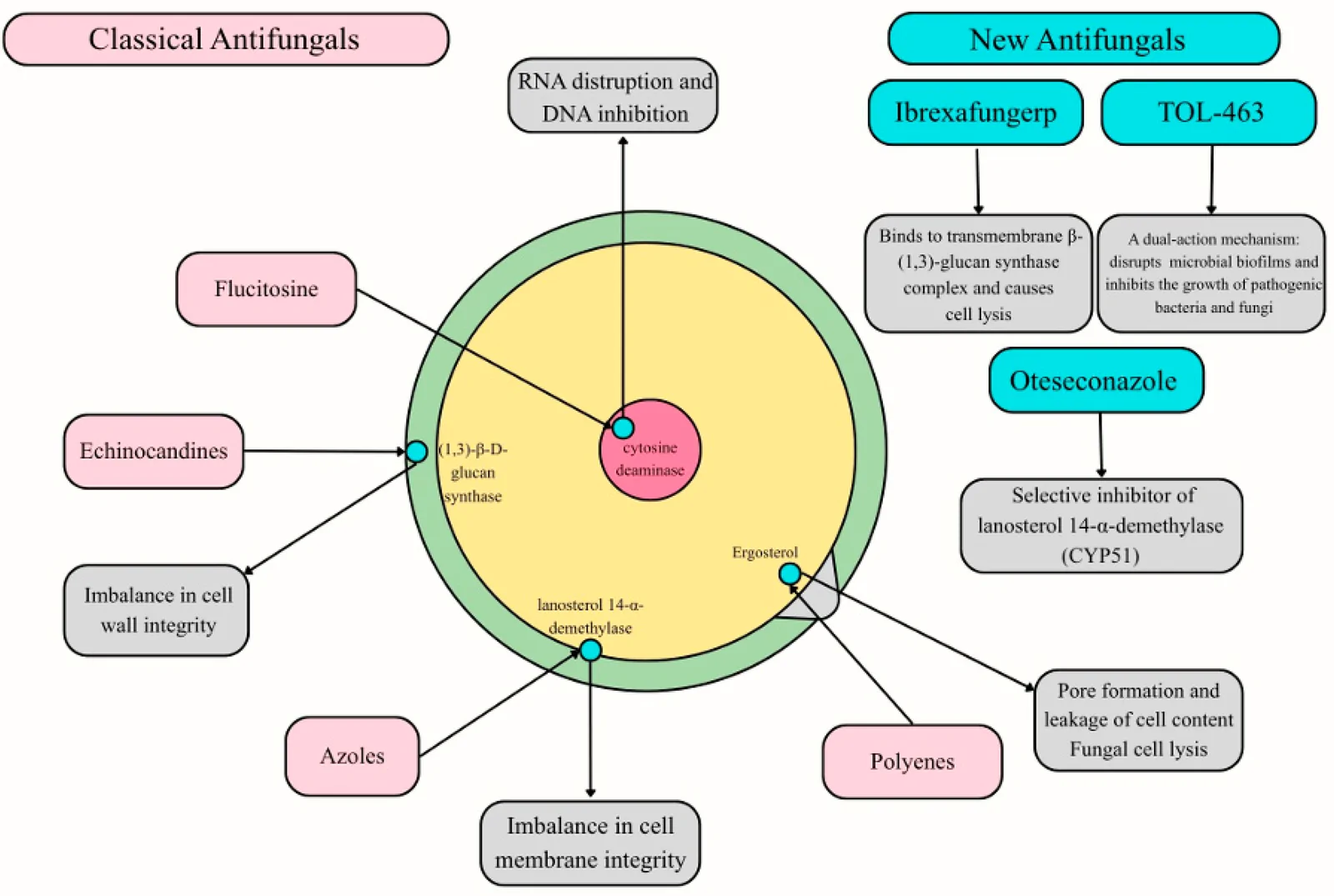
Overview of the mechanisms of action of classical and novel antifungal drugs considered for the treatment of VVC. Figure description: TOL-463, a novel vaginal anti-infective drug composed of boric acid and EDTA.

Among the causes of *C. albicans* resistance to antifungal agents, it is worth mentioning the overexpression of multidrug transporters—Mdr1 (multidrug resistance protein 1) from the MFS (major facilitator superfamily) protein family, and Cdr1p and Cdr2p (Candida drug resistance 1/2) from the ABC (ATP-binding cassette) protein family [75]. These proteins are responsible for the transport of many compounds, including azoles. The phenomenon of increased expression of these pumps is therefore of particular importance in the development of resistance to this class of drugs [70,75]. Drug resistance is an important virulence factor of *C. albicans*. Upon exposure to the drug, the fungi increase the secretion of hydrolytic enzymes (such as SAP proteases and LIP lipases) and enhance their adhesive properties, which increases their invasiveness [71,75]. An additional challenge in treatment is the significantly increased tolerance to the drugs used when a biofilm forms [69]. The mechanisms of action of classical and new antifungal drugs considered in the therapy of VVC are presented in Figure 2.

### 5.1. Treatment Recommendations

For the treatment of uncomplicated VVC in healthy, non-pregnant women and mild symptoms—most commonly caused by *C. albicans*—the following topical (intravaginal) therapy is used: clotrimazole (e.g., 100 mg for 7 days, 200 mg for 3 days or 500 mg as a single dose), miconazole (e.g., 1 application daily for 3–7 days), econazole, sertaconazole, and tioconazole—alternative formulations [69,75]. The list of optional treatments provided by ISSVD additionally includes fenticonazole, econazole and isoconazole [30]. For systemic (oral) treatment, usually applied in severe cases, fluconazole 150 mg is administered as a single dose or itraconazole 200 mg two times for 3 days [26,70]. The efficacy of topical and systemic treatment is comparable and exceeds 80% [71]. A modern oral alternative for the treatment of acute VVC is ibrexafungerp (300 mg twice daily for one day), which demonstrates high efficacy even in azole-resistant cases [69,78].

Complicated VVC (e.g., severe symptoms, infection in patients with diabetes or immunosuppression, infection with a species other than *C. albicans*) requires more intensive treatment. In cases of severe symptoms, two or three doses of fluconazole 150 mg administered every 72 h are recommended [69,70]. For recurrent candidiasis in such patients, the standard approach includes long-term maintenance therapy. The standard of care is induction therapy with fluconazole (e.g., 150 mg every 72 h—3 doses) followed by maintenance therapy: fluconazole 150 mg once weekly for 6 months [26,30,69,70,79]. In the case of fluconazole-resistant strains or when fluconazole cannot be used for other reasons, an alternative therapeutic option is long-term topical clotrimazole therapy (1% cream 5 g nightly for 14 days followed by 5 g vaginally twice a week for 6 months) [26]. It is worth emphasising again that initiating such aggressive, long-term therapy should be preceded by microbiological testing and pathogen species identification. Similarly, control cultures carried out after the completion of therapy would be very helpful in assessing its effectiveness.

An innovative option for preventing RVVC recurrences in women who are not of childbearing age is oteseconazole, administered on a weekly schedule for 11 weeks following the induction phase [69,70,80]. As previously mentioned, this drug is currently not available in Europe.

In cases of infection with *N. glabratus*, which exhibits naturally reduced susceptibility to azoles, treatment is based on the intravaginal administration of boric acid (600 mg daily for 14–21 days) or nystatin (100,000 IU for 14 nights) [69,70]. In cases resistant to these methods, off-label combination therapy remains an option: intravaginal flucytosine (17%) with amphotericin B [69,81].

Treatment of VVC during pregnancy must be limited exclusively to topical vaginal preparations (clotrimazole, miconazole, or nystatin for 7 days), as oral fluconazole is associated with a risk of miscarriage and foetal malformations [70,82,83]. Boric acid, like oteseconazole and ibrexafungerp, is contraindicated during pregnancy due to potential teratogenicity [69,70,84].

Sexual partners generally do not require routine treatment unless they exhibit symptoms such as balanitis [69]. Although studies confirm the possibility of sexual transmission of the same *Candida* strains, routine treatment of asymptomatic partners does not reduce the incidence of recurrent infections in women [85,86,87].

Additional support for treatment, particularly in cases of recurrence, involves maintaining the health of the vulvar skin by using emollients instead of soap and using water-based lubricants during intercourse, which helps prevent microtraumas that trigger yeast overgrowth [69]. Research is also underway on new strategies, such as zinc supplementation, which inhibits the expression of *C. albicans* virulence factors, e.g., zinc-binding protein Pra1 associated with local inflammation [69,88]. Table 4 summarises basic information regarding treatment.

### 5.2. New Anti-Candida Therapeutic Alternatives

Given the growing multidrug resistance of *Candida* species to standard azole drugs, plant-derived compounds represent a promising therapeutic alternative for the treatment of VVC [89]. Phytochemicals, like antifungal drugs, are characterised by a multi-faceted mechanism of action, which includes, amongst other things, the destabilisation of fungal cell membranes, the induction of oxidative stress through the accumulation of reactive oxygen species, and the inhibition of key virulence factors, such as adhesion and biofilm formation [90,91]. The main groups of compounds active against fungi are polyphenols, terpenes and alkaloids, but other plant-derived compounds are also being investigated. Polyphenols (including flavonoids and tannins) are one of the most numerous groups of secondary metabolites under investigation. Quercetin, rutin and gallic acid have been shown to damage the cell membrane, inhibit biofilm formation and exhibit synergism with fluconazole [89,90]. Extracts rich in flavonoids, e.g., from *Fridericia chica*, effectively inhibit the transformation of yeasts into an invasive filamentous form, thereby impairing the pathogen’s ability to penetrate epithelial tissues [90,92]. Terpenes and terpenoids, found, amongst other sources, in essential oils, act primarily by destabilising the cytoplasmic membrane and disrupting mitochondrial function [89,90]. Carvacrol and triterpenoids from the fruit of *Solanum torvum* (e.g., betulinic and oleanolic acids) exhibit potent inhibitory activity against fluconazole-resistant strains [89,90]. Berberine, an alkaloid, inhibits the adhesion of *C. albicans* to vaginal epithelial cells and inhibits biofilm formation [89,92]. Another example of an alkaloid is chelerythrine, which disrupts the structure of the biofilm and induces cell death via oxidative stress [89,90]. Many plant compounds and extracts are being investigated for use in the treatment of VVC. Curcumin, derived from *Curcuma longa*, blocks ergosterol synthesis and inhibits the secretion of proteases [89,92]. Clinical trials have shown that curcumin-based creams may be more effective at relieving itching and treating infections than clotrimazole [89,92]. Allicin from *Allium sativum* significantly reduces the expression of virulence genes responsible for the transition of yeasts to an invasive filamentous form [92]. Cannabidiol exhibits a complex mechanism of action, including the destruction of biofilm by reducing the expression of genes responsible for cell wall synthesis and drastically lowering ATP levels in fungal cells [89,92]. Chebulinic acid from *Terminalia chebula* has been shown in in silico studies to exhibit greater inhibitory activity against *C. albicans* growth than standard fluconazole [89]. Dill seed oil (*Anethum graveolens*) destroys the fungus’s mitochondria and inhibits mitochondrial dehydrogenase activity, leading to cell death [89,92]. Key considerations regarding the use of phytotherapeutics include their stability, tissue penetration and bioavailability, as well as their toxicity at the concentrations at which they exhibit antifungal activity. A brief summary of plant-derived compounds is presented in Table 5.

### 5.3. Probiotics in the Treatment of Vulvovaginal Mycoses (VVC)

The goal of using probiotics in VVC is to restore the normal vaginal microbiome, particularly the dominance of lactobacilli (*Lactobacillus* spp.), which inhibit the growth of *Candida*, lower the vaginal pH through the production of lactic acid (55–111 mM), compete for adhesion to the epithelium, and strengthen local immunity [69,93]. Some strains, particularly *Lactobacillus crispatus*, have been shown to inhibit the adhesion of *C. albicans* to epithelial cells [69]. Furthermore, specific small molecules produced by *Lactobacillus* species can block a key virulence factor—the formation of pseudohyphae (filaments)—by inhibiting a kinase from the DYRK1 family [9].

The most commonly used probiotic strains for VVC (vaginal or oral):

- Vaginal: *Lactobacillus crispatus*
- Oral/vaginal: *Lactobacillus rhamnosus* GR-1
- Oral/vaginal: *Lactobacillus reuteri* RC-14
- Oral: *Lactobacillus acidophilus*
- Oral: Multistrain mixtures (*L. plantarum*, *L. fermentum*, *Bifidobacterium*)

In the treatment of acute VVC, probiotics are not effective as monotherapy and do not replace antifungal treatment. Although they are often advertised as preventive or adjunctive measures, studies with a low risk of bias do not recommend their routine use [69]. The unsatisfactory and inconsistent results of probiotic therapy may stem from the fact that the introduction of additional strains does not permanently restore the optimal composition of the microbiome [69].

Nevertheless, when used in combination with antifungal medications, probiotics may improve the effectiveness of treatment and reduce the risk of recurrence, especially when combined with maintenance therapy [70]. Clinical studies have shown, for example, that supplementation with preparations containing *Saccharomyces cerevisiae*, melatonin, and *Lactobacillus acidophilus* GLA-14 in combination with clotrimazole results in higher clinical efficacy and fewer recurrences compared to the use of an antifungal drug alone [94]. Research is also underway on the use of oral probiotics to prevent recurrent infections in pregnant women [95].

Currently, probiotics are often used as complementary therapy; however, there are no clear recommendations. The efficacy of commercially available products depends heavily on the properties of the specific strain, the route of administration (oral vs. vaginal), and the duration of use [69]. Due to inconsistent clinical trial data, international guidelines (e.g., IDSA, CDC) do not recommend the routine use of probiotics in the treatment of VVC [30,69,70].

### 5.4. New VVC Therapies

Over the past five years, several experimental therapies for vulvovaginal candidiasis have been developed and approved, particularly for cases of recurrent vaginal candidiasis. NDV-3A is an experimental vaccine based on the Als3 protein, which is essential for the adhesion of *Candida albicans* to the epithelium. In clinical trials, vaccination with this preparation doubled the percentage of patients without recurrence of infection compared to the control group. Phase 2 clinical trials of the NDV-3A vaccine have been completed for two main indications, and the preparation is awaiting the start of registration (Phase 3) trials [96,97].

TOL-463 is a vaginal preparation containing boric acid and EDTA, which exhibits antifungal and antibacterial activity and improves treatment efficacy by disrupting microbial biofilms. Clinical trials in women with RVVC and bacterial vaginosis have demonstrated high efficacy (81–92%) and safety. The product is currently in the clinical trial phase [98].

## 6. Summary

This review indicates that vulvovaginal candidiasis, particularly its recurrent form, poses a significant clinical, social, and economic challenge on a global scale. Although *Candida albicans* remains the predominant etiological agent, there has been an increase in the prevalence of non-albicans species, such as *Nakaseomyces glabratus* and *Pichia kudriavzevii*, which is associated, among other factors, with the selective pressure exerted by commonly used azole medications. The pathogenesis of the infection is multifactorial, resulting from complex interactions between host factors—such as microbiome dysbiosis, hormonal fluctuations, diabetes, or genetic predisposition—and sophisticated fungal virulence mechanisms, including the ability to form biofilms and secrete cytotoxic candidalysin. In the short term, the priority is to raise diagnostic standards in order to avoid therapeutic errors and reduce selective pressure on resistant strains. Therefore, priority should be given to developing and evaluating diagnostic tools that allow rapid detection of *Candida* as well as assessing microbiota composition, especially in mixed infections and distinguishing between beneficial and pathogenic microbiomes (e.g., *Lactobacillus iners).* Furthermore, antifungal susceptibility testing can still cause difficulties, so adjustment to the existing methodology may be needed. The uncontrolled use of over-the-counter antifungal medicines leads to misdiagnoses (e.g., confusing VVC with bacterial vaginosis) and the development of resistance and tolerance in fungi. The use of induction and maintenance regimens remains the standard of care for RVVC, ensuring control of the infection in high-risk patients, such as women with diabetes or compromised immunity. In the longer term, the widespread introduction of innovative medicines into clinical practice and a clearer definition of the role of complementary medicine will be crucial. Modern antifungal drugs should demonstrate high efficacy against azole-resistant strains and represent a breakthrough in the treatment of RVVC. Probiotics and phytotherapy also appear to play an important role as complementary therapy; therefore, more research in this area is needed, given the inconclusive results obtained so far. Although international guidelines do not recommend their routine use as monotherapy, probiotics may help restore microbiome homeostasis. Research into plant-derived compounds is paving the way for new, multi-pronged therapeutic strategies. Promoting good hygiene, zinc supplementation, and the use of emollients provide significant support in preventing micro-injuries to the epithelium, which facilitate colonisation. Long-term strategies focus on the complete elimination of recurrences through biological interventions and a deeper understanding of host–pathogen interactions. Research into vaccines, such as NDV-3A, offers hope for a significant prolongation of symptom-free periods and lasting protection against *Candida* infections. The introduction of preparations such as TOL-463, designed to physically destroy the structure of the fungal biofilm—which is the main cause of persistent and recurrent infections on the vaginal epithelium—also appears to be key.

In summary, combating VVC requires a shift from empirical symptomatic treatment to precision medicine based on species-specific diagnosis, modern pharmacotherapy and innovative preventive methods, such as vaccination.

## Figures and Tables

**Table 1:** Most important factors affecting vaginal health.

Factor	Effect	Impact on *Candida*	Clinical Outcome
High Oestrogen Levels	Stimulates glycogen deposition in the vaginal epithelium.	Glycogen serves as a carbon source for yeasts, promoting proliferation.	Increased susceptibility to VVC (e.g., during pregnancy or hormone replacement therapy).
Hyperglycaemia	Elevates glucose concentrations in mucosal secretions.	Promotes massive yeast proliferation, increased adhesion, and biofilm formation.	Higher incidence of VVC and RVVC.
Lactobacillus Metabolites	Production of lactic acid (55–111 mM), short-chain fatty acids, and lactate.	Lowers vaginal pH and directly inhibits the production of invasive hyphae.	Homeostasis: *C. albicans* remains in the non-invasive blastospore form.
Zinc Supplementation	Introduction of exogenous zinc.	Inhibits the expression of *C. albicans* virulence factors.	Potential therapeutic support to reduce infection severity.
Antibiotics/Douching	Elimination of beneficial *Lactobacillus* species.	Leads to dysbiosis, removing inhibitory metabolites (lactic acid) and allowing *Candida* outgrowth.	Development of symptomatic VVC.

**Table 2:** Epidemiology of vaginal candidiasis in selected publications from the last five years.

References	Region/Country	Data	Population Tested	VVC Frequency	Etiology	Remarks
Jacob et al., 2018 [56]	Germany	2014–2016	General (women in gyneacological practices) 1000 pts	5.3%	No data	Patients treated with Clo—72% FLU—14% NYS—6%
Mbatudde et al., 2025 [57]	Uganda	2023	HIV -positive 144 pts	49%	*C. albicans*—62% NCAC—37.6%	
Aboagye et al. 2025 [58]	Ghana (Ho, Volta Region)	2020–2023	pregnant women 205 pts	50.7%—out of them 47% symptomatic	*C. albicans*—49/ 53% * C. dubliniensis—2/5.5% * C. krusei—36.7/20% * N. glabratus—12/ 23.6% * C. tropicalis—0/9% *	55% *C. albicans* resistant to FLU
Roy et al., 2024 [59]	North-Eastern India		120 pregnant women, including 60 asymptomatic	35% (42/120) 45% (27/60) in symptomatic & 25% (15/60) in asymptomatic	*C. albicans*—37/53.3% * NCAC—62.95/46.6% * *N. glabratus*—37.5% *C. parapsilosis*—25% *C. tropicalis*—20.8% *C. krusei*—16.67%	16.6% *C. albicans* and 37.5% NCAC resistant to FLU
Ismael SS., 2024 [60]	Iraq		400 vaginal samples from symptomatic women	27.5%—*Candida* positive		2.5% mixed *Candida- Trichomonas* infection
Kroustali et al., 2025 [61]	Greece	2019–2021	1300 adult women with vaginitis	18% (233/1300)	*C. albicans*—50% *C. parapsilosis*—35% *N. glabratus*—10% *Candida* spp.—5%	4% *C. albicans* and 23% *N. glabratus* resistant to FLU
Maraki et al., 2019 [62]	Greece	2012–2017	10,256 women with vaginitis	11.9% *Candida* positive 5.1%—RVVC	*C. albicans*—75.6% *N. glabratus*—13.6%	6.6% resistant to FLU
Pereira et al., 2021 [63]	Brazil		278 pts (173 symptomatic and 105 asymptomatic)	53.2% of symptomatic pts, 12.4% asymptomatic	*C. albicans*—80.9% NCAC—17.1% *Rhodotorula mucilaginosa*—1%	
Benedict et al., 2022 [64]	United States		4548 women invited to survey, out of them 1869 respond	5.2% had VVC in the past year, out of them 4.7% had RVVC	No data	The study estimated self-reported incidence
Yano et al., 2019 [17]	United States	2016–2018	284 non-pregnant women	77.5% VVC (34.6% RVVC) 22.5% without history of VVC	No data	Study based on online survey information
Church et al., 2025 [65]	Canada	2015–2018	293,853 tests	11% (32,894)	No data	Analysis performed on the basis of publicly available datasets
Ratner et al., 2025 [66]	United Kingdom, Leeds	2018–2021	5461 samples from women with complicated or RVVC	Culture positivity 33.5% (1828)	Yearly prevalence of: *C. albicans*—94; 90.7; 87.4%, NCAC—6; 9.3; 12.6% *N. glabratus*—2.8; 5.5; 6.8%	Yearly percentage of FLU-resistant *C. albicans*—0.4; 1.5, and 2.19%
Bowen et al., 2025 [67]	Ecuador	2021–2022 (12-moths)	195 symptomatic, non-pregnant women	36.4% (71/195)—including 15 (7.7%) RVVC	*C. albicans—90%, of whom 14% were* in mixed infections. *C. albicans* in 60–80% RVVC *N. glabratus*—8%	8% *C. albicans* FLU-resistant
Gubran et al. 2026 [68]	Jemen	2023 (3 months)	102 symptomatic women	39.2% (40/102)	*C. albicans*—55%, *C. krusei*—17.5%, *N. glabratus*—12.5%, *C. tropicalis*—10% *C. parapsilosis*—5%	4.5% *C. albicans* FLU-resistant

Description: Values marked with (*) indicate the percentages of particular species in groups of patients with/without symptoms of vaginal infection. NCAC—Non-Candida albicans Candida; Clo—clotrimazole; FLU—fluconazole; NYS—nystatine.

**Table 3:** Comparison of methods applied in diagnosis of VVC [26,30,69,70,71,72,73,74].

Diagnostic Tools	Characteristics	Advantages	Limitations
Physical examination	Crucial investigation	The assessment of clinical symptoms; the basis for implementing differential diagnosis and empirical treatment	Risk of false diagnosis (under-, over- or misdiagnosis); should be confirmed by laboratory tests (at least direct microscopy)
Laboratory Diagnostic			
Direct microscopy: 10% KOH; calcofluor white, Gram staining;	Sensitivity 40–70%	inexpensive, rapid, detection of hyphae/pseudohyphae suggests infection	does not identify the species
Commercial NAAT/PCR tests	Validated tests can be used in diagnosis of VVC	Rapid, allows detection and identification of fungi in vaginal samples	Sensitivity and specificity depend on the test; usually do not include rare yeast species
Culture	Gold standard; mandatory in refractory cases, or with clinical symptoms and negative microscopy	Allows subsequent species identification and susceptibility testing	Takes 2–4 days to obtain final results Required to establish diagnosis of RVVC and cVVC
Chromogenic media		Reduce time to final result; help detect multispecies infections; high specificity in *C. albicans* species complex differentiation	Morphology of some NCAYs similar to *N. glabratus*. Cannot be used as sole test for *N. glabratus* identification.
Commercial metabolic tests for yeast identification, sometimes coupled with AFST (automated systems or manual)		Identify most common pathogenic yeast species	24–48 h incubation required; sensitivity/specificity depend on test construction and database updates; rare species could be missed
MALDI-TOF MS		Rapid and highly specific method	Sensitivity/specificity database-related, requires specialized equipment
Molecular identification of isolated yeasts	ITS1-2 Sequencing	Gold standard	Not routinely available
Antifungal susceptibility: Microdilution methods, disc diffusion/E-test methods	MIC values	Allows guiding treatment; recommended in recurrent and refractory infection	No clinical breakpoints for topical drugs. Vaginal pH affects the efficacy of topical antifungal agents; need to develop standards tailored to this niche

**Table 4:** Treatment strategies for vulvovaginal infections caused by *Candida* spp. (VVC/RVVC) according to global guidelines [69,70].

Clinical Category	Recommended Treatment Regimen	Notes and Specific Guidelines
Uncomplicated VVC (nonpregnant women, most commonly *C. albicans*)	Oral treatment: Fluconazole 150 mg (single dose). Topical treatment: Clotrimazole (500 mg once or 100 mg for 7 days), miconazole (3–7 days).	The efficacy of topical and systemic treatment is comparable and exceeds 80%. Alternatives: econazole, sertaconazole, tioconazole.
RVVC and complicated forms (severe symptoms, diabetes, immunosuppression)	Induction phase: Fluconazole 150 mg every 72 h (on days 1, 4, and 7). Maintenance phase: Fluconazole 150 mg once weekly for 6 months.	RVVC is defined as ≥4 episodes within 12 months. In women past childbearing age, oteseconazole is an option.
Non-albicans species (e.g., *N. glabratus*)	Boric acid: 600 mg vaginally once daily (14–21 days). Nystatin: 100,000 IU vaginally (14 nights).	These species exhibit naturally reduced susceptibility to azoles. In refractory cases: off-label therapy (17% flucytosine + amphotericin B).
Treatment during pregnancy	Topical preparations only: Clotrimazole, miconazole, or nystatin used for 7 days.	Strictly contraindicated: oral fluconazole (risk of foetal malformations/miscarriages), boric acid, oteseconazole, ibrexafungerp.
Modern treatment options	Ibrexafungerp: 300 mg twice daily for one day. Oteseconazole: Weekly regimen (monotherapy or sequentially with fluconazole).	Ibrexafungerp is a glucan synthase inhibitor effective against azole-resistant strains.
Additional recommendations	Sexual partners: Treat only if symptomatic (e.g., balanitis). Supplementation: Studies on zinc.	Routine treatment of asymptomatic sexual partners does not reduce the recurrence rate in women. The use of emollients is recommended.

**Table 5:** Plant-sourced compounds as new therapeutic anti-*Candida* candidates.

Phytochemicals/Plant-Derived Compounds	Mechanism of Action	Key Findings
Polyphenols (quercetin, rutin, gallic acid)	Damage to the cell membrane and inhibition of biofilm formation	The synergistic effect when combined with fluconazole
Flavonoids (e.g., from *Fridericia chica*)	Inhibiting the transformation of yeast cells into an invasive filamentous form	A reduction in the pathogen’s ability to penetrate epithelial tissues.
Terpenes and terpenoids	Destabilisation of the cytoplasmic membrane and disruption of mitochondrial function	Carvacrol and triterpenoids (e.g., betulinic acid) are effective against FLU-resistant strains
Berberine (alkaloid)	Inhibition of adhesion to vaginal epithelial cells and inhibition of biofilm formation	Preventing fungal colonisation and growth
Chelerythrine (alkaloid)	Destruction of the biofilm structure and induction of cell death via oxidative stress.	Causing a rapid increase in reactive oxygen forms
Curcumin	Inhibition of ergosterol synthesis and suppression of protease secretion	In clinical trials, it is more effective at relieving itching than clotrimazole
Allicin	Downregulation of virulence genes responsible for the filamentous form	Inhibiting a key stage in the transition to the invasive form
Cannabidiol (CBD)	A reduction in the expression of cell wall synthesis genes and a significant decrease in ATP levels	Leading to the destruction of the biofilm and the energy exhaustion of the cell
Chebulinic acid	Strong inhibition of *C. albicans* growth (demonstrated in silico studies)	Higher efficacy than standard fluconazole
Fennel seed oil	Destruction of mitochondria and inhibition of mitochondrial dehydrogenase activity	Causing the direct death of fungal cells

## Data Availability

No new data were created or analysed in this study. Data sharing is not applicable to this article.

## Abbreviations

The following abbreviations are used in this manuscript:

VVC	Vulvovaginal Candidiasis
RVVC	Recurrent Vulvovaginal Candidiasis
cVVC	chronic Vulvovaginal Candidiasis
NCAY	Non-*Candida Albicans* Yeast
HSCT	Haematopoietic Stem Cell Transplant
TLR	Tool-Like Receptor
IL	Interleukin
IFN-γ	Interferon gamma
NK	Natural Killer cells
HS	heparan sulfate
MBL2	mannose-binding lectin 2
Als	Agglutinin-like sequence
Hwp1	Hyphal-specific cell wall protein 1
PMN	polymorphonuclear neutrophiles
NLRP3	NOD-like receptor protein 3 inflammasome
EPS	extracellular polymeric substances
ABC	ATP (adenosine-triphosphoran)-binding cassette (a protein family)
CDR	Candida drug resistance
MFS	major facilitator superfamily
Mdr	multidrug resistance protein
Hsps	heat shock proteins
QSMs	quorum-sensing molecules
MLST	multilocus sequence typing
PCR	Polymerase chain reaction
HIV	human immunodeficiency virus
RNA	ribonucleic acid
DNA	deoxyribonucleic acid
ROS	Reactive Oxygen Species
Clo	clotrimazole
FLU	fluconazole
NYS	nystatine
AMB	amphotericin B
5-FC	5-fluorocytosine, flucytosine
NCAC	non-*Candida albicans Candida*
MALDI-TOF MS	matrix-assisted laser desorption/ionization time-of-flight mass spectrometry
FDA	United States Food and Drug Administration
SAP	secreted aspartic protease
ASM	American Society for Microbiology
ECMM	European Confederation of Medical Mycology
ISHAM	International Society for Human and Animal Mycology
ISSVD	International Society for the Study of Vulvovaginal Disease
EMA	European Medicines Agency
IDSA	Infectious Diseases Society of America
CDC	Centers for Disease Control
NAAT	Nucleic acid amplification test
TOL-463	a novel vaginal anti-infective drug composed with boric acid and EDTA
EDTA	Ethylenediaminetetraacetic acid
